# Secukinumab treatment demonstrated high drug survival and sustained effectiveness in patients with severe chronic plaque psoriasis: 21‐month analysis in Australian routine clinical practice (SUSTAIN study)

**DOI:** 10.1111/ajd.13895

**Published:** 2022-07-09

**Authors:** Peter Foley, Nick Manuelpillai, Con Dolianitis, Geoffrey D. Cains, Eric Mate, Rebecca Tronnberg, Christopher Baker

**Affiliations:** ^1^ Skin Health Institute Carlton Victoria Australia; ^2^ The University of Melbourne, St Vincent's Hospital Melbourne Fitzroy Victoria Australia; ^3^ Royal Melbourne Hospital Parkville Victoria Australia; ^4^ Liverpool Hospital Sydney New South Wales Australia; ^5^ Novartis Pharmaceuticals, Australia Pty Limited Macquarie Park New South Wales Australia

**Keywords:** Australia, drug survival, durability, psoriasis, real‐world and clinical setting, secukinumab

## Abstract

**Background:**

Drug survival measures the rate and duration of adherence to a given therapeutic agent and evaluates its long‐term effectiveness, safety, and real‐world utility. The SUSTAIN study sought to establish the drug survival and effectiveness of secukinumab for patients with severe chronic plaque psoriasis (CPP) in the Australian clinical setting.

**Methods:**

Data of all patients (aged ≥18 years) from Australasian Psoriasis Registry (APR) treated with secukinumab were analysed. The primary objective was to describe the drug survival of secukinumab at 9 months. Key secondary objectives included drug survival of secukinumab at 3, 6, 15, and 21 months, stratified by biologic‐naïve vs biologic‐experienced patients; proportion of patients achieving Psoriasis Area and Severity Index (PASI) 75/90/100 responses; and changes in health‐related quality of life over time utilising the Dermatology Life Quality Index (DLQI).

**Results:**

Of 294 patients included in this analysis, 110 (37.4%) were biologic‐naïve and 184 (62.6%) biologic‐experienced. Kaplan–Meїer drug survival rates in biologic‐naïve vs biologic‐experienced patients were 0.92 vs. 0.86 (9 months) and 0.82 vs. 0.68 (21 months), respectively. The proportion of patients with PASI 75/90/100 responses for biologic‐naïve vs. biologic‐experienced was 100/87.7/38.4 vs 98.5/61.5/27.2 (9 months) and 100/81.0/41.7 vs. 98.4/62.0/24.2 (21 months), respectively. The mean (standard deviation [SD]) DLQI in biologic‐naïve vs. experienced patients was 2.2 (4.1) vs. 3.1 (5.2) (9 months) and 1.4 (2.5) vs. 3.1 (5.3) (21 months). No new safety signals were observed.

**Conclusions:**

Secukinumab demonstrated high drug survival and sustained effectiveness in Australian real‐world setting, in biologic‐naïve and biologic‐experienced patients with severe CPP.

## INTRODUCTION

Psoriasis is an immune‐mediated chronic inflammatory disease, characterised by well‐defined red plaques with silver or white scales that may be itchy and vary in severity.^1^ Along with physical discomfort, psoriasis has a significant impact on patient quality of life.^2^, ^3^ Psoriasis affects 2%–4% of the population in Western countries,^4^ with a prevalence of 2.3%–6.6% in Australia.^5^

Secukinumab is a fully human monoclonal antibody that selectively neutralises interleukin‐17A and has proven to be highly effective in the long‐term treatment of the multiple manifestations of psoriatic disease, including psoriasis localised to the nails, scalp, palms, soles, and joints.^6^, ^7^, ^8^, ^9^, ^10^ It was approved in Australia for the treatment of moderate‐to‐severe chronic plaque psoriasis (CPP) on 12th January 2015 and was listed under the Pharmaceutical Benefits Scheme on 1st September 2015.

Drug survival is the rate and duration of adherence to a given therapeutic agent. It can be an indirect measure of long‐term real‐world effectiveness and safety of the therapy.^7^ Results of drug survival rate studies of biologics for psoriasis differ with study design and population.^7^, ^8^ Consequently, real‐world studies have shown conflicting results for the drug survival of secukinumab in patients with psoriasis.^12^, ^13^, ^14^ In a real‐world setting involving Spanish patients, secukinumab showed consistent effectiveness and drug survival results, independent of patient status of biologic‐naïve or biologic‐experienced.^2^ A clinical study conducted over a 5‐year period demonstrated high and sustainable levels of skin clearance following treatment with secukinumab, along with a favourable safety profile in patients with moderate to severe CPP.^10^, ^12^ Routine clinical practice differs from country to country, and no real‐world data have been published on the drug survival of secukinumab in patients with severe CPP within the Australian clinical setting.

The purpose of SUSTAIN study was to establish the drug survival rate and effectiveness of secukinumab in the treatment of patients with severe CPP in the Australian routine clinical setting.

## METHODS

### Objectives

The primary objective of the SUSTAIN study was to describe the drug survival rate of secukinumab in patients with severe CPP at 9 months after initiating treatment. Secondary objectives of the study included descriptive demographics and clinical characteristics of patients initiating treatment with secukinumab and the survival rate of secukinumab at months 3, 6, 15, and 21, stratified by biologic‐naïve and biologic‐experienced patients. Real‐world effectiveness of secukinumab, with respect to the proportion of patients achieving whole body Psoriasis Area and Severity Index (PASI) 75/90/100 responses, changes in health‐related quality of life over time using the Dermatology Life Quality Index (DLQI), and the use of concomitant medication in addition to secukinumab was also assessed as secondary endpoints.

### Study design and patient population

SUSTAIN was a retrospective, non‐interventional, cohort study that used secondary data from the Australasian Psoriasis Registry (APR). The APR is an online database that collects clinical data from people suffering with psoriasis in Australia and New Zealand.^15^ Data of patients from Australia (aged ≥18 years) who initiated secukinumab (300 mg) treatment between 12th January 2015 and 31st December 2019, for severe CPP (PASI >15), were extracted retrospectively from the APR. The extracted data were transferred to a contract research organisation (Datalytics Pty Ltd) for analysis. Only patients with data for each applicable time point were included in the analysis for that time point.

The extracted study population was stratified into patients without and with prior biologic exposure (biologic‐naïve and biologic‐experienced, respectively), at the initiation of treatment with secukinumab. The biologic‐experienced group was subcategorised based on the number of biologic agents used (1, 2 or ≥3) before the first use of secukinumab.

### Assessments

**FIGURE 1:**
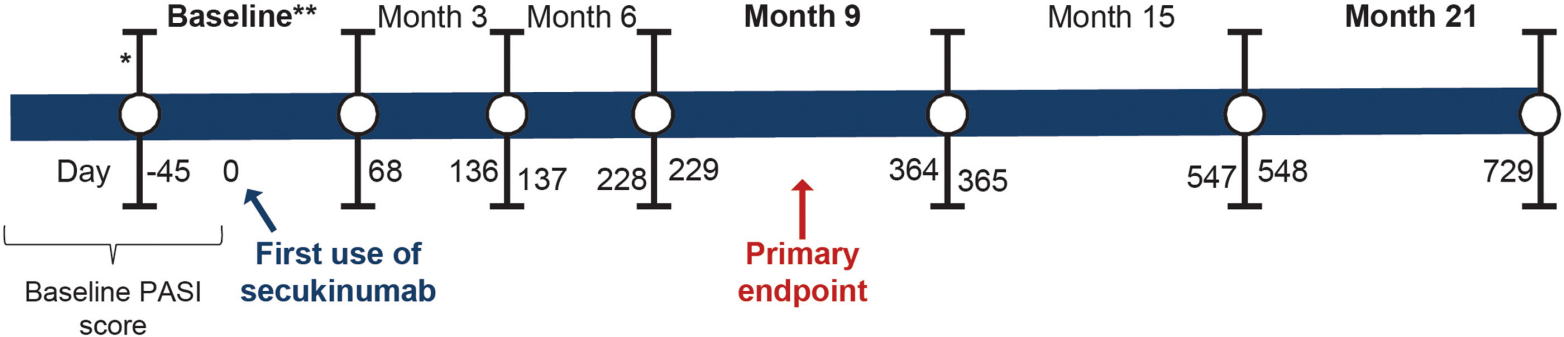
SUSTAIN study design. *Depending on the patient, the time interval between baseline (start of the treatment cycle that included the first dose of secukinumab) and Day 0 may have included periods of treatment with other biologics. **Time interval between Day −45 and Day 0. Abbreviation: PASI, Psoriasis Area and Severity Index.

Patients were analysed for PASI and DLQI at baseline and at months 3, 6, 9, 15, and 21 in accordance with the routine clinical practice in Australia. Day 0 was the time point when secukinumab was first used. The time points for assessing PASI were relative to Day 0. Baseline was defined as the start of the treatment cycle that included the first use of secukinumab and may have differed from Day 0 based on patients having different treatment cycles and having used other biologics before secukinumab. Not all patients had a PASI assessment at Day 0; hence, PASI at baseline was calculated as the mean of PASI recorded between Day −45 and 0 (Figure 1).

### Statistical analyses

Kaplan–Meier survival curves were used to examine drug survival patterns. The analyses were performed using STATA statistical software. Missing PASI and DLQI were imputed with the last observation carried forward. The Overall Patient population set included patients in the dataset extracted from the APR. Full Analysis Set (FAS) included all patients from the APR who satisfied the inclusion criteria described in study design and patient section. The PASI Analysis Set (PAS) comprised patients in the FAS excluding those with no whole body PASI data. The DLQI Analysis Set (DAS) included patients from the FAS excluding those with no DLQI data.

## RESULTS

### Patient disposition

Data of 301 patients were extracted from the APR. Of these, 294 (97.7%) patients were included in the FAS. The remaining seven patients (2.3%) received the first dose of secukinumab before 12th January 2015 and were excluded from the study. Of the 294 secukinumab‐treated patients who were included in this analysis, 110 (37.4%) were biologic‐naïve, and 184 (62.6%) were biologic‐experienced. Of the 184 biologic‐experienced patients, 94 (32.0%), 42 (14.3%), and 48 (16.3%) patients had previous exposure to 1, 2, and ≥3 biologics, respectively (Figure S1).

### Demographics and baseline disease characteristics

Demographics and patient characteristics at baseline were assessed for 281 patients (FAS minus 13 patients who did not have a recorded consultation visit before their first use of secukinumab) at Day 0. Of these, 59.4% of patients were male. Mean age of overall patients was 48.4 years; mean weight was 90.7 kg, and mean body mass index was 30.8 kg/m^2^. The mean duration of CPP was 20.8 years, 29.6% of patients had a prior PsA diagnosis, and 59.9% had a reported family history of CPP. In total, 234 (83.3%) patients had whole body affected by CPP, with the scalp being the most frequently affected body region (45.6%). The baseline demographics and disease characteristics of biologic‐naïve and biologic‐experienced patients were consistent with the findings of overall patients, with few exceptions (Table 1). In total, 217 patients (77.2%) had ≥1 comorbidity, with obesity (62.7%) being the most frequently reported, followed by hypertension (31.6%) and depression (28.7%).

**TABLE 1:** Baseline demographics and disease characteristics

Characteristics^**a**^	FAS (*n* = 281)	Biologic‐naïve (*n* = 100)	Biologic‐experienced			
			Overall (*n* = 181)	1 Prior biologic (*n* = 93)	2 Prior biologics (*n* = 41)	≥3 Prior biologics (*n* = 47)
Gender, *n* (%)						
Male	167 (59.4)	59 (59.0)	108 (59.7)	60 (64.5)	22 (53.7)	26 (55.3)
Female	114 (40.6)	41 (41.0)	73 (40.3)	33 (35.5)	19 (46.3)	21 (44.7)
Age (years)	48.4 (12.8)	46.4 (13.4)	49.5 (12.3)	50.4 (13.0)	49.2 (9.9)	48.0 (12.8)
Weight (kg)	90.7 (22.9)	90.5 (21.9)	90.9 (23.4)	90.1 (25.3)	90.3 (20.2)	93.0 (22.3)
BMI (kg/m^2^)	30.8 (6.6)	30.0 (6.5)	31.2 (6.6)	30.8 (6.6)	31.1 (6.3)	32.0 (6.8)
Smoking status, *n* (%)						
Current smoker	46 (16.4)	11 (11.0)	35 (19.3)	17 (18.3)	7 (17.1)	11 (23.4)
Former smoker	56 (19.9)	17 (17.0)	39 (21.5)	22 (23.7)	9 (22.0)	8 (17.0)
Non‐smoker	179 (63.7)	72 (72.0)	107 (59.1)	54 (58.1)	25 (61.0)	28 (59.6)
Time since diagnosis of plaque psoriasis (years)	20.8 (12.0)	15.9 (10.9)	23.3 (11.8)	20.7 (12.4)	25.8 (11.0)	25.4 (11.0)
PASI (whole body)						
At Day 0	13.0 (10.5)	20.5 (8.7)	8.5 (8.8)	9.6 (9.6)	8.9 (10.2)	6.6 (5.9)
At baseline	24.0 (8.0)	22.7 (7.5)	24.7 (8.1)	23.0 (7.3)	25.0 (8.1)	27.1 (9.0)
DLQI						
At Day 0	11.0 (9.1)	16.6 (8.8)	7.8 (7.7)	8.8 (8.8)	6.2 (6.0)	7.5 (6.8)

Abbreviations: BMI, body mass index; DLQI, Dermatology Life Quality Index; FAS, full analysis set; n, number of patients; PASI, Psoriasis Area Severity Index; SD, standard deviation.

^a^ All values are mean (SD), unless otherwise stated.

### Drug survival of secukinumab

**FIGURE 2:**
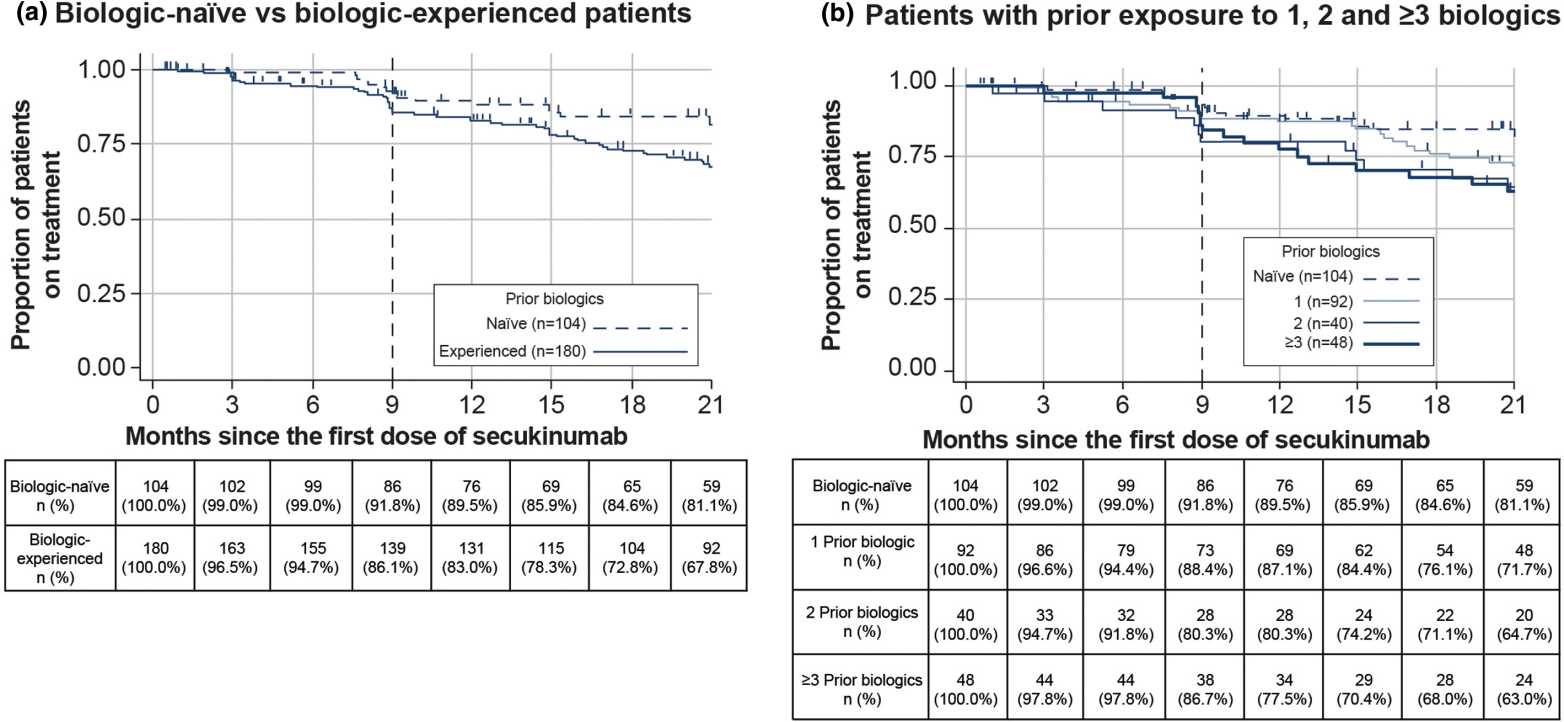
Kaplan–Meїer drug survival rate in (a) biologic‐naïve vs biologic‐experienced patients and (b) patients with prior exposure to 1, 2, and ≥3 biologics (full analysis set). 10 patients had no known follow‐up after starting secukinumab. Abbreviation: n, number of patients.

The Kaplan–Meier estimate of the proportion of overall patients who remained on secukinumab at 9 months after initiating treatment was 0.88 (95% CI: 0.837, 0.916). At 9 months, the drug survival rate in biologic‐naïve patients was higher than that in biologic‐experienced patients: 0.92 (95% CI: 0.843, 0.958) vs. 0.86 (95% CI: 0.798, 0.905), respectively (Figure 2a). Among the biologic‐experienced patients, at 9 months, the survival rates of patients treated with 1, 2, and ≥3 prior biologics were 0.88 (95% CI: 0.794, 0.936), 0.80 (95% CI: 0.630, 0.901), and 0.87 (95% CI: 0.727, 0.938), respectively (Figure 2b). The proportion of overall patients who remained on secukinumab at 3–21 months was as follows: Month 3: 0.97 (95% CI: 0.947, 0.988); Month 6: 0.96 (95% CI: 0.933, 0.980); Month 15: 0.81 (95% CI: 0.757, 0.854); and Month 21: 0.73 (95% CI: 0.667, 0.780). The proportion of biologic‐naïve vs biologic‐experienced patients and patients with prior exposure to 1, 2, and ≥3 biologics, who were treated with secukinumab at 3, 6, 15, and 21 months, is provided in Figure 2b.

### Real‐world effectiveness of secukinumab

#### Proportion of patients achieving whole body PASI 75/90/100 responses

**FIGURE 3:**
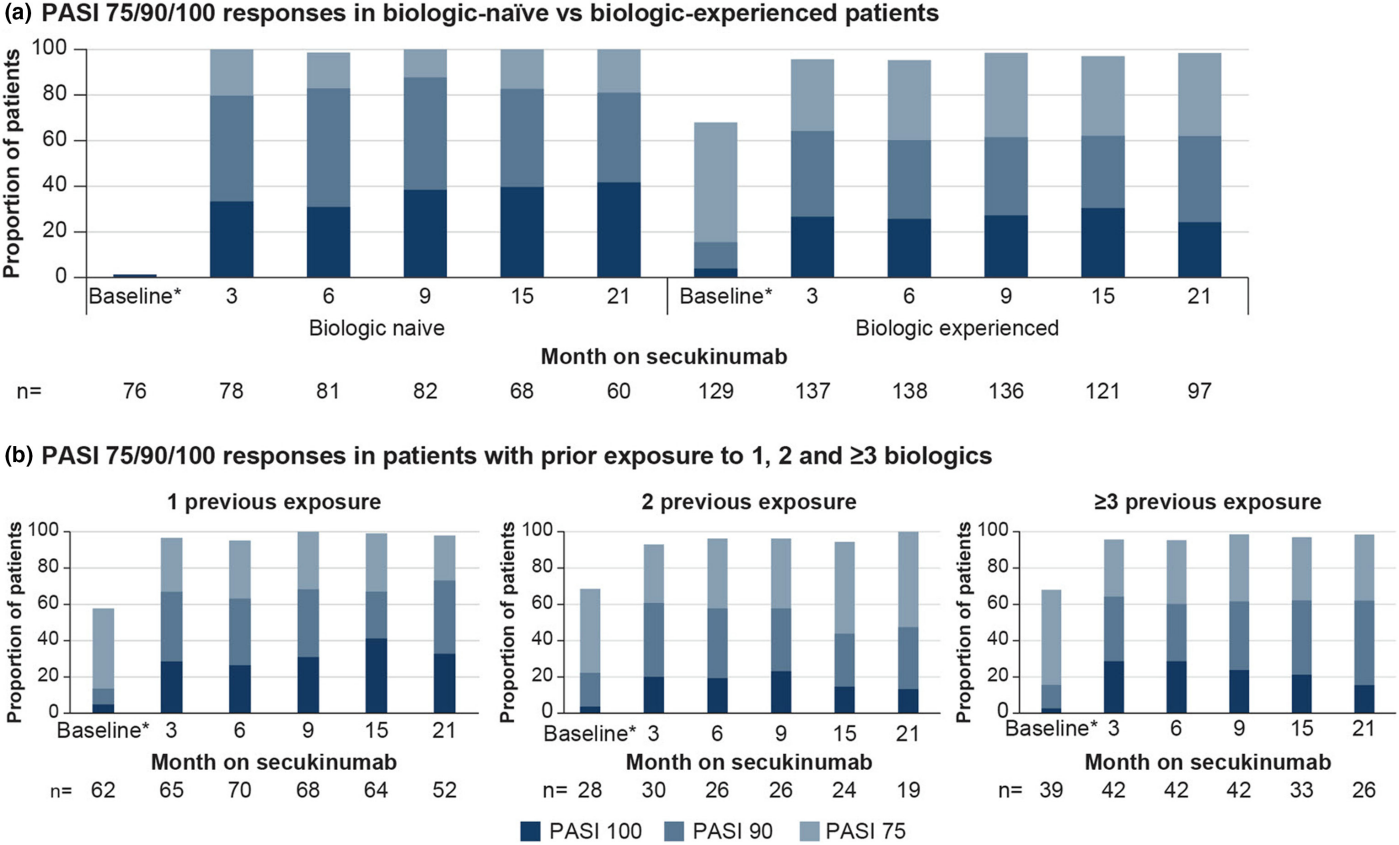
Whole‐body PASI 75/90/100 responses in (a) biologic‐naïve vs biologic‐experienced patients and (b) patients with prior exposure to 1, 2, and ≥3 biologics (PASI analysis set; last observation carried forward). *Time interval between Day −45 and Day 0. *n*, number of patients; PASI, Psoriasis Area and Severity Index.

In total, 262 patients were included in the PAS. Missing values were imputed with the last observation carried forward. The proportion of whole body PASI 75/90/100 responders of biologic‐naïve vs biologic‐experienced was PASI 75: 100.0% vs. 98.5% at Month 9 and 100% vs. 98.4% at Month 21; PASI 90: 87.7% vs. 61.5% at Month 9 and 81.0% vs. 62.0% at Month 21; and PASI 100, 38.4% vs. 27.2% at Month 9, and 41.7% vs. 24.2% at Month 21 (Figure 3a). The proportion of PASI responders by prior exposure to 1, 2, and ≥3 biologics is provided in Figure 3b.

#### Changes in health‐related quality of life over time using DLQI

**FIGURE 4:**
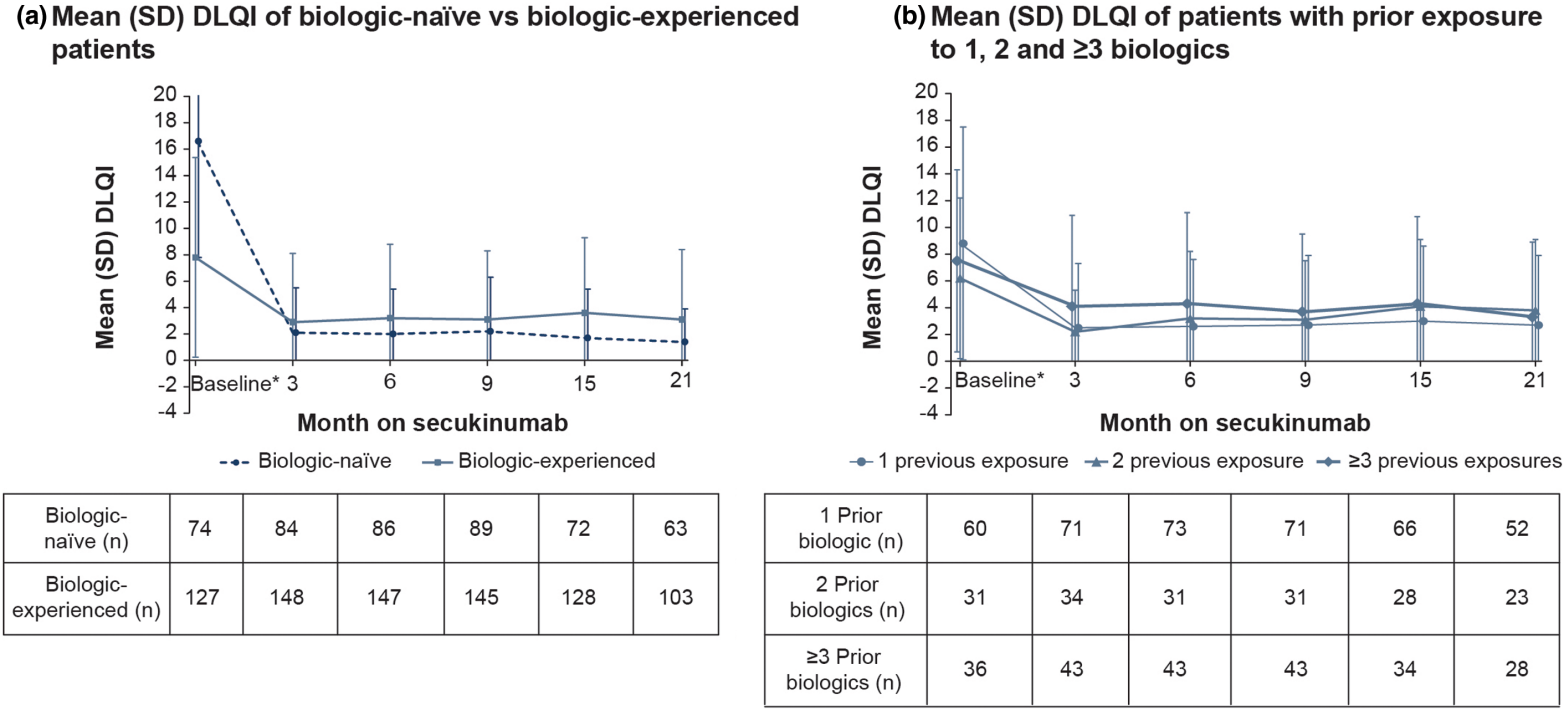
Mean DLQI of (a) biologic‐naïve vs biologic‐experienced patients and (b) patients with prior exposure to 1, 2, and ≥3 biologics (DLQI analysis set; last observation carried forward). *Time interval between Day −45 and Day 0. Abbreviations: DLQI, Dermatology Life Quality Index; n, number of patients; SD, standard deviation.

Overall, 277 patients were included in the DAS. The mean DLQI (SD) of biologic‐naïve vs biologic‐experienced patients recorded between Day 0 and Day −45 was 16.6 (8.7) vs. 7.8 (7.6), respectively. It decreased to 2.2 (4.1) vs. 3.1 (5.2) and 1.4 (2.5) vs. 3.1 (5.3) at Months 9 and 21, respectively. At Month 21, the mean (SD) DLQI was lower for biologic‐naïve than for biologic‐experienced patients (1.4 [2.5] vs. 3.1 [5.3], respectively; Figure 4a). For patients with prior exposure to 1, 2, and ≥3 biologics, the mean (SD) DLQI was lower for patients with previous exposure to 1 biologic than for patients with previous exposure to 2 or ≥3 biologics (Figure 4b).

### Use of concomitant medication

Topical corticosteroids were the most frequently used concomitant medications; eight (2.7%) patients started corticosteroids before the first use of secukinumab; 59 (20.1%) patients started corticosteroids on or after the first use of secukinumab and ended on or before the last known use of secukinumab; and 11 (3.7%) patients started corticosteroids on or after the first use of secukinumab and continued after the known end of secukinumab. Methotrexate was used in 11 (3.7%) patients before the first use of secukinumab, in 18 (6.1%) patients on or after the first use of secukinumab and ended on or before the last known use of secukinumab, and in 12 (4.1%) patients on or after the first use of secukinumab and continued after the known end of secukinumab use. The complete list of concomitant treatments is provided in Table S1.

### Safety

Overall, 70 adverse events were reported by 54 (18.4%) patients. The most common adverse events were infections requiring systemic antibiotics (*n* = 16; 5.4%), arthritis (*n* = 5; 1.7%), infections requiring topical antibiotics (*n* = 5; 1.7%), and conception (*n* = 5; 1.7%) (Table 2).

**TABLE 2:** Adverse events with onset during first treatment with secukinumab (FAS)

Adverse event^**a**^	Events	Patients
Any adverse event	70	54
Infections requiring systemic antibiotic, antifungal, or antiviral treatment(s)	18	16
Arthritis (including de novo and worsening of pre‐existing arthritis)	5	5
Infections requiring topical antibiotic, antifungal or antiviral treatment(s)	5	5
Conception ‐ Patient	5	5
Infections requiring hospitalisation	3	3
Other skin, hair, nail and subcutaneous disorder	3	3
Basal cell carcinoma	2	2
Anxiety	2	2
Birth ‐ Full term ‐ Patient	2	2
Other general disorder	2	2
Shingles	2	2

Abbreviations: APR, Australasian Psoriasis Registry; FAS, full analysis set.

^a^ The adverse event labels are the verbatim adverse event text as used in the APR. Instances of occurrence of more than one adverse event are reported here.

## DISCUSSION

SUSTAIN is the first real‐world study performed within Australian routine clinical practice to determine the drug survival of secukinumab in patients with severe CPP. Secukinumab demonstrated drug survival in a large proportion of patients up to Month 21 of the study. The sustained effectiveness was demonstrated by the proportion of patients who achieved whole body PASI 75/90/100 responses by Month 3 and maintained these responses throughout the study. The consistent improvements in the DLQI throughout the study also indicates a sustained response with secukinumab. These improvements were similar in patients without or with prior exposure to biologics. The decrease in drug survival over the 3‐ to 21‐month period on secukinumab was consistent with the secukinumab drug survival reported by a 52‐week real‐world evidence study conducted in Canada.^16^ The drug survival results for biologic‐naïve patients were relatively higher than that of biologic‐experienced patients at Months 9 and 21, indicating that biologic‐naïve patients adhered to secukinumab treatment longer than biologic‐experienced patients. Survival rates of secukinumab treatment were also higher for patients with 1 previous biologic exposure than for those with either 2 or ≥3. PASI 75/90/100 responses were higher in biologic‐naïve patients than with the biologic‐experienced at 9 and 21 months, indicating that biologic‐naïve patients had a better response to secukinumab treatment than with biologic‐experienced patients. As per Australian clinical practice, patients not responding to secukinumab would likely have been switched to another biologic. The difference between biologic‐naïve and biologic‐experienced patients might reflect differences in smoking history, which may affect the efficacy of biologics. A possible explanation is that smoking influences biologic efficacy or the tendency to swap biologics.^17^, ^18^ The DLQI was lower in biologic‐naïve patients than with the biologic‐experienced patients at 9 and 21 months, indicating a greater improvement in the quality of life of patients not exposed to prior biologic(s) than with those exposed to prior biologics.

These results are also in line with the data from BADBIR registry that represented long‐term effectiveness of secukinumab over 24 months in both biologic‐naïve and biologic‐experienced patients in a real‐world setting (UK). The study reported a drug survival function of 0.88 at Year 1, that was sustained up to Year 2 at 0.77,^13^ compared with SUSTAIN study, demonstrating a drug survival of 0.88 at Month 9 that was sustained up to Month 21 at 0.73.^13^The Dutch BioCAPTURE registry provides another example of drug survival of secukinumab. The study reported biologic‐naïve patients as having a 1‐year survival function of 0.90, compared with 0.74 survival function for biologic‐experienced patients.^14^, ^19^

As SUSTAIN was based on the secondary use of data, the safety analyses were provided on an aggregate level. The most frequent events were related to infections which is consistent with the secukinumab product label.^11^

Limitations of the study include comorbidity data being restricted to field names used in the APR, missing data points because most of the data fields are optional and the time lag between the patient consultation visit and their data being registered in the database. A potential bias may also have occurred due to PBS criteria, requiring patients to demonstrate a PASI 75 response to continue on government reimbursed therapy and due to the statistical method used to account for missing data (last observation carried forward).

## CONCLUSIONS

SUSTAIN was a non‐interventional, retrospective cohort study with a primary objective of establishing the drug survival rate and effectiveness of secukinumab in the treatment of patients with severe CPP in the Australian routine clinical setting. Secukinumab demonstrated high drug survival and sustained effectiveness in both biologic‐naïve and experienced patients.

Biologic‐naïve patients undergoing secukinumab treatment had better drug survival and effectiveness than with biologic‐experienced patients. No new safety signals were observed in the APR, and the safety profile of secukinumab was in line with its label.

## FUNDING INFORMATION

This investigation was sponsored by Novartis Pharmaceuticals Australia Pty Ltd.

## CONFLICT OF INTEREST

P. Foley has served on the advisory board and/or as a consultant/investigator and/or received research grant/speaker's honoraria/travel grants from Abbvie, Akaal, Amgen, Arcutis, Argenx, ASLAN, AstraZeneca, Boehringer Ingelheim, Botanix, Bristol Myers Squibb, Celgene, Celtaxsys, CSL, Cutanea, Dermira, Eli Lilly, EVELO Biosciences, Galderma, Genentech, GenesisCare, GSK, Hexima, Janssen, Kymab Ltd, Leo Pharma, Mayne Pharma, MedImmune, Melaseq/Geneseq, Merck, Novartis, Pfizer, Regeneron Pharmaceuticals Inc, Reistone Biopharma, Roche, Sanofi, Sun Pharma, Teva, UCB Pharma and Valeant. N. Manuelpillai is a sub‐investigator on trials involving Abbvie, ASLAN, AstraZeneca, Boehringer Ingelheim, Bristol Myers Squibb, Dermira, Eli Lily, Galderma, LEO, Pfizer, Reistone Biopharma, Sanofi, Sun Pharma and UCB. C. Dolianitis is a member of advisory committee and received grants from Abbvie, Janssen, Merck, Pfizer. G. D. Cains: Abbvie and Sun Pharma. E. Mate and R. Tronnberg are employees of Novartis Pharmaceuticals Australia Pty Ltd. C. Baker has served on the advisory board and/or as a consultant/investigator and/or received research grant/speaker's honoraria from Abbvie, Pfizer, Novartis, Janssen, Lily, and Leo.

## ETHICAL APPROVAL

The study protocol was approved by the Bellberry Ltd independent human research ethics committee (Application No: 2019‐11‐991). The study was conducted according to the Declaration of Helsinki.

## Supporting information


Table S1

Figure S1

Appendix S1

## References

[1] Johnson‐Huang LM , Lowes MA , Krueger JG . Putting together the psoriasis puzzle: an update on developing targeted therapies. Dis Model Mech. 2012;5:423–33. 10.1242/dmm.009092 22730473 PMC3380706

[2] Ruiz‐Villaverde R , Rodriguez‐Fernandez‐Freire L , Galán‐Gutierrez M , Armario‐HIta JC , Martinez‐Pilar L . Drug survival, discontinuation rates, and safety profile of secukinumab in real‐world patients: a 152‐week, multicenter, retrospective study. Int J Dermatol. 2020;59:633–9. 10.1111/ijd.14819 32173862

[3] Wasel N , Poulin Y , Andrew R , Chan D , Fraquelli E , Papp K . A Canadian self‐administered online survey to evaluate the impact of moderate‐to‐severe psoriasis among patients. J Cutan Med Surg. 2009;13:294–302. 10.2310/7750.2009.08066 19919806

[4] Parisi R , Symmons DP , Griffiths CE , Ashcroft DM . Global epidemiology of psoriasis: a systematic review of incidence and prevalence. J Invest Dermatol. 2013;133:377–85. 10.1038/jid.2012.339 23014338

[5] Subedi S , Gong Y , Chen Y , Shi Y . Infliximab and biosimilar infliximab in psoriasis: efficacy, loss of efficacy, and adverse events. Drug Des Devel Ther. 2019;13:2491–502. 10.2147/DDDT.S200147 PMC6661374 31413544

[6] Boehncke WH , Schön MP . Psoriasis. Lancet. 2015;386:983–94. 10.1016/S0140-6736(14)61909-7 26025581

[7] Augustin M , Jullien D , Martin A , Peralta C . Real‐world evidence of secukinumab in psoriasis treatment ‐ a meta‐analysis of 43 studies. J Eur Acad Dermatol Venereol. 2020;34:1174–85. 10.1111/jdv.16180 31919937

[8] van den Reek JM , Zweegers J , Kievit W , Otero ME , van Lümig PP , Driessen RJ , et al. 'Happy' drug survival of adalimumab, etanercept and ustekinumab in psoriasis in daily practice care: results from the BioCAPTURE network. Br J Dermatol. 2014;171:1189–96. 10.1111/bjd.13087 24807471

[9] Menter A , Papp KA , Gooderham M , Pariser DM , Augustin M , Kerdel FA , et al. Drug survival of biologic therapy in a large, disease‐based registry of patients with psoriasis: results from the Psoriasis Longitudinal Assessment and Registry (PSOLAR). J Eur Acad Dermatol Venereol. 2016;30:1148–58. 10.1111/jdv.13611 27027388 PMC5071685

[10] Bissonnette R , Luger T , Thaçi D , Toth D , Lacombe A , Xia S , et al. Secukinumab demonstrates high sustained efficacy and a favourable safety profile in patients with moderate‐to‐severe psoriasis through 5 years of treatment (SCULPTURE Extension Study). J Eur Acad Dermatol Venereol. 2018;32:1507–14. 10.1111/jdv.14878 29444376 PMC6175198

[11] Australian product information [Cosentyx]. Vol. 2021.

[12] Papp K , Gooderham M , Beecker J , Lynde CW , Delorme I , Dei‐Cas I , et al. Rationale, objectives and design of PURE, a prospective registry of patients with moderate to severe chronic plaque psoriasis in Canada and Latin America. BMC Dermatol. 2019;19:1–7. 10.1186/s12895-019-0087-3 31226985 PMC6588885

[13] Yiu ZZN , Mason KJ , Hampton PJ , Reynolds NJ , Smith CH , Lunt M , et al. Drug survival of adalimumab, ustekinumab and secukinumab in patients with psoriasis: a prospective cohort study from the British Association of Dermatologists Biologics and Immunomodulators Register (BADBIR). Br J Dermatol. 2020;183:294–302. 10.1111/bjd.18981 32124442

[14] Daudén E , de Lima G , Armesto S , Herrera‐Acosta E , Vidal D , Villarasa E , et al. Multicenter retrospective study of secukinumab drug survival in psoriasis patients in a daily practice setting: a long‐term experience in Spain. Dermatol Ther. 2021;11(6):2207–15. 10.1007/s13555-021-00606-9 PMC8611164 34561788

[15] Autralasian psoriasis registry. Vol. 2021.

[16] Georgakopoulos JR , Ighani A , Phung M , Yeung J . Drug survival of secukinumab in real‐world plaque psoriasis patients: a 52‐week, multicenter, retrospective study. J Am Acad Dermatol. 2018;78:1019–20. 10.1016/j.jaad.2017.11.036 29180095

[17] Ribeiro PÁ , Fonseca JE , Vieira‐Sousa E . Remission persistence in rheumatoid arthritis, psoriatic arthritis and axial spondyloarthritis under biologic treatment. Ann Rheum Dis. 2019;23:1027–8. 10.1136/annrheumdis-2019-eular.8034

[18] Walsh J , Amato D , Kerdel F , Krueger G . Differences in psoriasis severity and exposure to immunomodulatory therapies between cigarette smokers and nonsmokers: observations from the PSOLAR registry in patients with moderate to severe psoriasis. J Am Acad Dermatol. 2013;68:AB197. 10.1016/j.jaad.2012.12.815

[19] van den Reek J , van Vugt LJ , van Doorn MBA , van der Kraaij GE , de WJA K , GPH L , et al. Initial results of secukinumab drug survival in patients with psoriasis: a multicentre daily practice cohort study. Acta Derm Venereol. 2018;98:648–54. 10.2340/00015555-2900 29405245

