# Supplementary material for: Secukinumab treatment demonstrated high drug survival and sustained effectiveness in patients with severe chronic plaque psoriasis: 21‐month analysis in Australian routine clinical practice (SUSTAIN study)
Source: Australas J Dermatol. 2022 Jul 9;63(3):303–11. doi: 10.1111/ajd.13895 (PMC9543110; doi:10.1111/ajd.13895)
Supplement: Supplementary file 1 — Table S1 [file AJD-63-303-s002.docx]

| Treatment for severe CPP | Overlap with first secukinumab treatment course | | |
| --- | --- | --- | --- |
|  | Start before,  stop after | Whole period | Start during |
| Secukinumab (Cosentyx) | − | 284 | − |
| Topical corticosteroids | 8 | 59 | 11 |
| Daivobet (Calcipotriol/Betamethasone) | 7 | 57 | 6 |
| Methotrexate | 11 | 18 | 12 |
| Enstilar (Calcipitrol/Betamethasone) | − | 5 | 26 |
| Vitamin D analogues | − | 15 | − |
| NBUVB: Whole body | 5 | 1 | 7 |
| Tar | 1 | 7 | 3 |
| Acitretin | − | 1 | 7 |
| Other: Topical | − | 2 | 4 |
| Corticosteroids 2 | − | 4 | 1 |
| Dithranol | 2 | 2 | − |
| Ustekinumab (Stelara) | 1 | 3 | − |
| Keratolytics | − | 3 | − |
| Topical tacrolimus | − | 1 | 2 |
| Emollients | − | 2 | − |
| Prednisolone | 1 | 1 | − |
| Prednisone | − | 1 | 1 |
| Adalimumab (Humira) | − | 1 | − |
| Folic Acid | 1 | − | − |
