# Supplementary figures and images for: Secukinumab treatment demonstrated high drug survival and sustained effectiveness in patients with severe chronic plaque psoriasis: 21‐month analysis in Australian routine clinical practice (SUSTAIN study)
Source: Australas J Dermatol. 2022 Jul 9;63(3):303–11. doi: 10.1111/ajd.13895 (PMC9543110; doi:10.1111/ajd.13895)

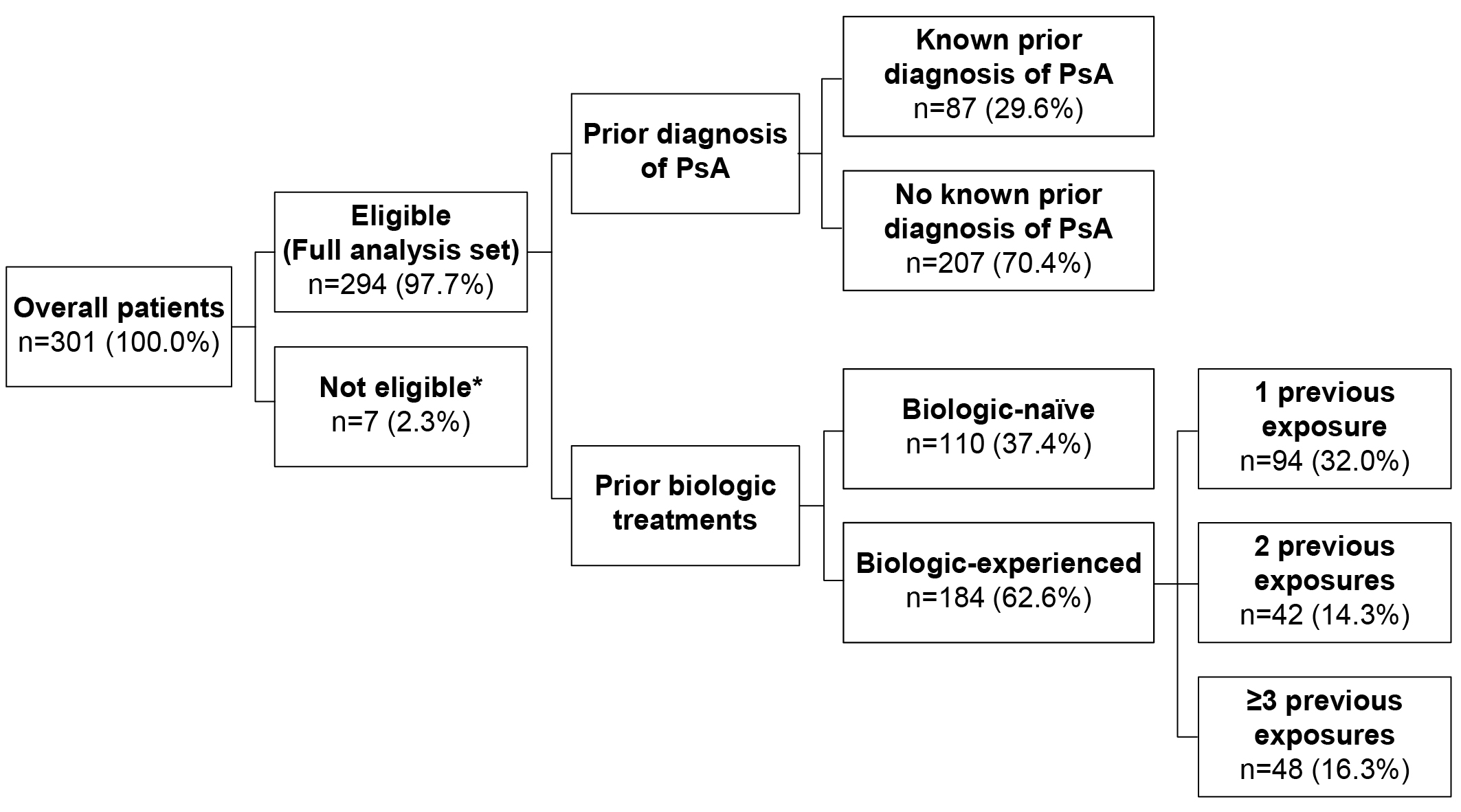

Supplement: Supplementary file 2 — Figure S1 [file AJD-63-303-s001.jpg]
