# Supplementary material for: Secukinumab treatment demonstrated high drug survival and sustained effectiveness in patients with severe chronic plaque psoriasis: 21‐month analysis in Australian routine clinical practice (SUSTAIN study)
Source: Australas J Dermatol. 2022 Jul 9;63(3):303–11. doi: 10.1111/ajd.13895 (PMC9543110; doi:10.1111/ajd.13895)
Supplement: Supplementary file 3 — Appendix S1 [file AJD-63-303-s003.docx]

# LEGENDS OF SUPPLEMENTARY TABLE AND FIGURE

Supplementary Table 1. Concomitant treatments for severe CPP (Full analysis set)

‘Start before, stop after’: treatments that begin before first use of secukinumab. ‘Whole period’: treatments that begin on or after first use of secukinumab and end on or before last known use of secukinumab. ‘Start during’: treatments that start on or after first use of secukinumab and which are continuing after known end of secukinumab. Concomitant treatments may be under-reported. Verbatim treatment text (TREATMENT), as reported in Australasian Psoriasis Registry. This table summarises treatment episodes that overlap the first secukinumab treatment course. Ten patients with no known duration of treatment with secukinumab are omitted from this table. Each treatment episode is classified by overlap with the first secukinumab course; patients may have more than one treatment episode.

CPP, Chronic Plaque Psoriasis.

Supplementary Fig. 1: Patient disposition

*Seven patients were not eligible for the study as they received the first dose of secukinumab before 12^th^ January 2015.
n, number of patients; PsA, psoriatic arthritis.
